# Regional Virtual Acute Care Helpline in Singapore at a National University Health System Virtual Care Centre: Retrospective Study

**DOI:** 10.2196/86556

**Published:** 2026-05-13

**Authors:** Linda Wang, Soon Hoe Ho, Mei Fong Liew, Serene Xin Lin Wong, Amartya Mukhopadhyay, Rachel Hui Choe, Priyanka Khatri, Nastar Ashna, Jonathan Edward Jacob, Tze Chin Wong, Teresa Koh, Lipin Tan, Si Hui Lee, Wern Lunn Kueh, Erna Gondo Santoso, Shikha Kumari, Satya Pavan Kumar Gollamudi

**Affiliations:** 1 Department of Medicine, Integrated Medicine Programme, Alexandra Hospital, National University Health System, Singapore, Singapore, 65 94247906; 2 Department of Medicine, Division of Respiratory and Critical Care Medicine, National University Hospital, National University Health System, Singapore, Singapore; 3 Department of Medicine, Yong Loo Lin School of Medicine, National University of Singapore, Singapore, Singapore; 4 Department of Medicine, Division of Advanced Internal Medicine, National University Hospital, Singapore, Singapore; 5 Department of Medicine, Ambulatory Operations, Alexandra Hospital, Singapore, Singapore; 6 Department of Medicine, Community, Alexandra Hospital, Singapore, Singapore; 7 Department of Medicine, Medical Affairs Office, Alexandra Hospital, Singapore, Singapore; 8 Department of Medicine, Nursing, Alexandra Hospital, Singapore, Singapore; 9 Department of Medicine, Academic Informatics Office, National University Health System, Singapore, Singapore

**Keywords:** emergency service, hospital, helpline, prehospital triage, emergency department, ED avoidance, community health services

## Abstract

**Background:**

Emergency department (ED) overcrowding and delayed access to care are ongoing challenges in Singapore. The COVID-19 pandemic further underscored the need for scalable virtual care models that go beyond traditional hospital settings, allowing patients to access acute specialist care quickly and efficiently.

**Objective:**

This study describes the design, implementation, and early outcomes of the National University Health System (NUHS) Virtual Care Centre (VCC), a clinician-led helpline aimed at reducing unnecessary ED visits and supporting community-based acute care.

**Methods:**

In 2020, the NUHS launched the VCC, a helpline at Alexandra Hospital, as a prehospital triage model. The helpline functions as a nurse-led telephone triage with real-time escalation to doctors for urgent medical issues. It ensures the continuity of care for patients recently discharged and diverts nonemergency cases from the ED. A retrospective analysis of call data from 2020 to 2024 was conducted to evaluate utilization patterns, clinical outcomes, and safety.

**Results:**

Over 4 years, the VCC managed 4857 calls, of which 59.3% (n=2879) were clinical in nature. Nearly two-thirds (1834/2879, 63.7%) were resolved remotely, preventing in-person ED visits. Only 13.8% (397/2879) required redirection to an ED, and 3.3% (95/2879) were directly admitted to an acute hospital or hospital at home service. Within 72 hours of call resolution, 69.1% (1990/2879) of the callers avoided an ED visit. Undertriage was 4.9% (110/2232) at 72 hours post call resolution, with no high dependency or intensive care unit admissions during this period. Mortality rates were low (1.0% at 14 days; 2.3% at 30 days).

**Conclusions:**

The NUHS VCC provides a feasible and safe model for virtual acute care triage within the public health care system. It effectively diverted lower-acuity cases from the ED and ensured continuity of care, offering a scalable approach aligned with national efforts to extend health care beyond hospital walls.

## Introduction

Overcrowding in emergency departments (EDs) is worsened by many patients seeking care for nonemergency health issues [1-3]. This increasing demand results in longer wait times, delays in critical treatments, and greater strain on hospital resources, ultimately affecting patient safety and clinical outcomes [4-6]. In Singapore, ED overcrowding has worsened over the years [7,8], partly due to an aging population, an increase in chronic diseases [9,10], and persistently high inpatient bed occupancy [11]. The data from the Singapore Civil Defence Force show a 22.1% rise in medical call volumes from 159,479 in 2021 to 194,722 in 2022, with a significant proportion of calls directed to EDs [12]. Such demand, combined with prolonged hospital stays for complex patients, contributes to high access block and delays in care downstream [8,11,13]. Addressing this challenge requires alternative care pathways that can safely manage lower acuity cases outside the ED.

To reduce ED overcrowding, health systems worldwide have explored virtual urgent care models, many of which accelerated during the COVID-19 pandemic [14]. These services offer alternatives to in-person ED visits by providing telephone or video consultations for patients with urgent but noncritical conditions [15-17]. For instance, Denmark’s MH-1813 helpline [18], a 24/7 telephone triage service for nonemergency cases, facilitates referrals to various acute services via a shared electronic medical record (EMR) system. Australia’s Victorian Virtual Emergency Department [19] provides video consultations with emergency clinicians for lower acuity symptoms. Similarly, Queensland’s Metro North Virtual ED [20] allows general practitioners (GPs), ambulance crews, and residential care providers to escalate uncertain cases to emergency clinicians. Canada’s Ontario Virtual Urgent Care (VUC) pilot [21,22] enables patients to consult with ED physicians via video. Other models, such as Spain’s Quirónsalud Virtual Urgent Care Program [23] and NHS 111 in England [24], use structured telephone or digital triage linked to regional health services. In the United States, Kaiser Permanente’s virtual urgent care model [25] connects to both primary and specialty care, facilitating scalable and timely responses to unscheduled care needs.

Despite these advances, most virtual emergency models remain episodic, focusing on short-term diversion from EDs. Key limitations include inconsistent handovers, weak integration with primary or longitudinal care, and limited engagement with community-based services [19-23]. Evaluations of Ontario’s VUC [21] reported fragmented follow-up, and Australia’s Victorian Virtual Emergency Department [19] lacked formal care transitions. In more structured systems, such as NHS 111 [24] and Kaiser Permanente [25], postconsultation coordination is not consistently documented or evaluated.

The National University Health System (NUHS) is a public health care cluster that primarily serves residents living in Singapore’s western region, comprising 3 acute hospitals, 7 polyclinics (large primary care centers with multiple primary care physicians), and a network of GP clinics [26]. In 2020, the NUHS launched the Virtual Care Centre (VCC) at Alexandra Hospital (AH) as a prehospital triage hotline to address service fragmentation and the increasing demand for acute care. Located within the NUHS cluster, the VCC supports continuity of care and diverts nonemergency cases from the hospital. The VCC core objectives are to (1) reduce unnecessary ED attendances through tele-triage and right-siting of care; (2) enable timely delivery of acute care in the community by collaborating with providers across nursing homes, primary care, and regional health teams; (3) reduce inpatient bed demand by deploying mobile care teams capable of providing hospital-level treatment at home (ie, NUHS@Home program, a hospital-at-home service) [27], or nursing interventions at homes (ie, first responders program); and (4) minimize interhospital transfers by directing patients to the most appropriate facility within the cluster.

Although virtual ward and hospital-at-home models have been reported locally [28,29], no prior evaluations of a regional acute care helpline in Singapore have been published. This paper provides the first detailed description of the NUHS VCC model and its early outcomes as a proof-of-concept. We describe the practical implementation of the VCC, including its design, operational workflow, and integration within the health system, and evaluate preliminary service utilization and safety indicators.

## Methods

### Conception and Implementation of the VCC Pilot Model

The NUHS VCC was piloted at AH in July 2020, during the peak of the COVID-19 pandemic, as a dedicated telephone helpline for postdischarge patients within the hospital (Figure S1 in Multimedia Appendix 1). These patients received predischarge printed brochures outlining when to call the VCC and SMS reminders 1 to 2 days post discharge. The service encouraged patients to contact the VCC for nonemergency health concerns rather than returning to the ED.

In 2021, the VCC expanded its services to include all inpatients, specialist outpatients, and ED attendees at AH. A structured protocol-based workflow was introduced, beginning with care managers (CMs), who conducted the initial triage of incoming calls using a 4-tier urgency protocol adapted from ED triage systems (Table 1 and Table S1 in Multimedia Appendix 2) [30,31]. Callers with medical issues were then referred to VCC physicians for further assessment either immediately or within a clinically appropriate timeframe. Physicians retained the discretion to adjust the urgency classification based on clinical judgment. Clinical management involved telephone or video consultations, leading to outcomes ranging from reassurance and advice to direct admission, urgent specialist referral, or first-responder nurse deployment. All encounters were documented within the hospital’s EMR system, facilitating communication across clinical teams. The VCC service operated on weekdays (8 AM-5:30 PM) throughout this phase.

**Table 1.** National University Health System (NUHS) Virtual Care Centre’s (VCC) 4-tier triage system for telephone-based acuity assessment^a^.

Escalation tier	Direct to	Time frame to first contact	Time frame to close case (decide on disposition and plan)	Escalation (last point)
Tier 1: inform patient to call 995	995 or the nearest emergency department	Immediate	Immediate	Note: There is no need to clear escalation with a doctor
Tier 2: urgent	Nursing or doctors	15 minutes: acknowledgment 45 minutes: call back	3 hours	Doctors
Tier 3: requires attention	Nursing	15 minutes: acknowledgment 45 minutes: call back	3 hours	Doctors
Tier 4: requires response	Nursing	4 hours	24 hours	Nursing

a Tier 1 (emergent) cases were immediately directed to emergency services (995/ED). Tier 2 cases required urgent review by VCC clinicians. Tiers 3 and 4 represented moderate- to low-acuity concerns, which were appropriate for teleconsultation. Initial triage was performed by care managers using protocolized scripts and red-flag criteria.

By September 2023, the VCC extended its prehospital triage services to NUHS-affiliated primary care providers. GPs within the NUHS Primary Care Network and physicians from the National University Polyclinics can consult VCC physicians in real time for clinical guidance and triage decisions, thereby allowing timely interventions and helping to prevent avoidable ED visits and hospital admissions.

In 2024, the VCC was scaled beyond AH to include additional NUHS-affiliated entities, such as NUHS@Home and Advanced Internal Medicine at National University Hospital [32]. A key improvement during this phase was the incorporation of nurses into the triage process (the original model only involved CMs and physicians), alongside CMs and physicians, thereby improving clinical assessment capacity and resolving cases at first contact.

### VCC Workflow

**Figure 1.**
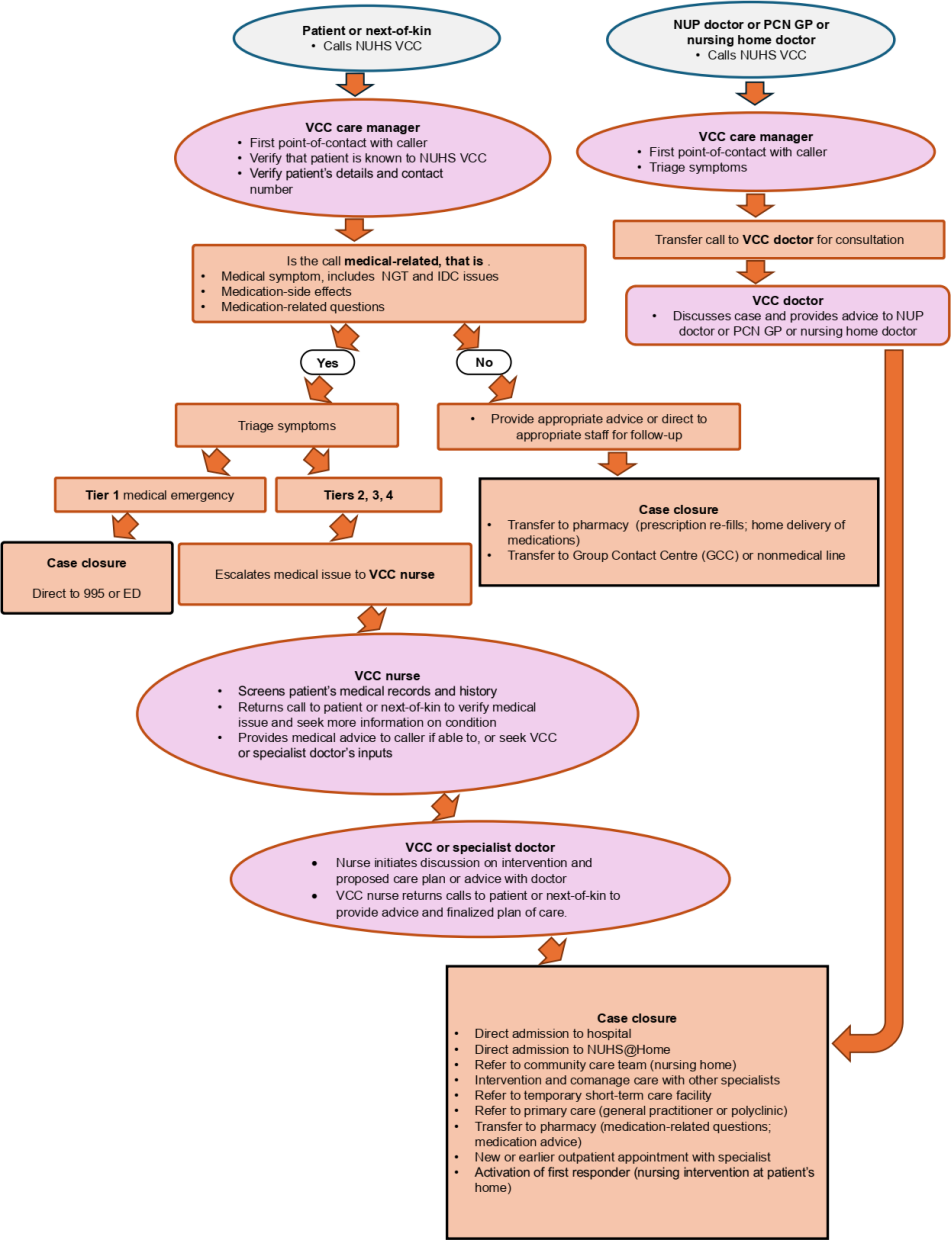
National University Health System (NUHS) Virtual Care Centre (VCC) model and workflow. This diagram illustrates the end-to-end virtual acute care pathway at Alexandra Hospital (AH), encompassing patient referral, eligibility screening, triage by care managers (CMs), and teleconsultation by nurses or doctors. It includes escalation protocols and coordinated follow-up. The system is integrated with electronic medical records (EMRs) and dispatch systems to enable seamless care delivery across providers. During the pilot phase, the VCC team comprised 2 CMs and 1 physician. The multidisciplinary team was later expanded to include CMs, emergency-trained nurses, and physicians across multiple specialties, including general medicine, geriatric medicine, general surgery, and orthopedic surgery. ED: emergency department; GP: general practitioner; IDC: indwelling catheter; NGT: nasogastric tube; NOK: next-of-kin; NUP: National University Polyclinics; PCN: primary care network.

The operational workflow of the VCC is both multidisciplinary and digitally integrated (Figure 1). CMs are the first point of contact and screen calls using standardized scripts. Nonmedical queries are redirected to the appropriate administrative office or contact center. For medical concerns, the CMs inform the caller that a health care provider will return their call shortly and then forward the call to the VCC’s clinical team. CMs also conduct follow-up calls approximately 1 to 2 weeks after the initial medical calls to assess the patient’s condition, collect feedback, and monitor service quality and patient satisfaction.

VCC nurses are experienced in acute care and manage most cases through telephone or video consultations. They collect medical histories, assess current symptoms, and determine the appropriate course of action. Nurses resolve many cases, while complex or ambiguous presentations are escalated to VCC physicians for further evaluation and assessment. VCC doctors, typically trained in acute medicine or relevant specialties, provide teleconsultations, prescribe medications, order investigations, coordinate referrals, and arrange hospital admissions when necessary. The team also provides clinical guidance to GPs and other referring clinicians, as well as coordinates access to services such as NUHS@Home and specialist outpatient clinics within the NUHS network.

The VCC workflow is supported by a modular digital infrastructure designed to facilitate timely triage, documentation, and coordination across care teams. A dedicated telephony system handles incoming and outgoing calls, while Microsoft apps (eg, Teams, Outlook) facilitate internal handovers, virtual team huddles, and communication across clinical teams. Clinical documentation is seamlessly integrated into the hospital’s EMR, ensuring that care decisions are accessible across teams and institutions. Dispatch coordination tools support the timely activation of mobile services, including nurse deployments and direct admissions to NUHS@Home. Central to the VCC model are the principles of “right-siting” and the “no wrong door” approach. The former ensures patients receive care at the most appropriate level, while, the latter obliges the VCC to assist all callers, even those falling outside its immediate scope, by connecting them to the correct service or helpline. This model reinforces the VCC’s role as an accessible entry point for urgent but nonemergency care in the region.

NUHS VCC began as a prehospital tele-triage pilot at AH and, over the course of 4 years, evolved into an integrated, cluster-wide service supporting urgent care across NUHS institutions and services. This expansion reflects both the model’s scalability and a deliberate strategy to embed virtual care into the cluster’s operational and digital infrastructure. In doing so, the VCC has begun to widen access to urgent care beyond the hospital walls.

### Data Collection and Analysis

We performed a retrospective review of all VCC medical call records from July 2020 (service launch) to December 2024. Data on call volume, call characteristics (medical vs. nonmedical), dispositions, and patient outcomes were extracted from NUHS’ Customer Relation Management record and NUHS VDO Office, Academic Informatic Office. Key outcome measures included ED avoidance (no ED presentation within 72 hours after the call), triage accuracy (matched, over, and under triage categories based on subsequent ED disposition), and adverse outcomes (intensive care unit [ICU] admissions within 72 hours and mortality at 14 and 30 days postcall). Patient satisfaction was assessed via follow-up telephone surveys in February and October to November 2024. Continuous variables are presented as means with SDs, and categorical variables as frequencies and percentages. No inferential statistical testing was performed. This study is reported in accordance with the STROBE (Strengthening the Reporting of Observational Studies in Epidemiology) guidelines.

### Ethical Considerations

This study involved a retrospective analysis of routinely collected operational data from the NUHS VCC. The study was submitted to the National Healthcare Group Domain Specific Review Board for the determination of ethics requirements. The board determined that the study did not require a formal ethics review as it involved the use of anonymized data and did not meet the definition of human subject research (NHG DSRB Ref: 2025‐0317). No identifiable personal data were accessed for the purpose of this study. The study was conducted in accordance with relevant institutional policies and guidelines.

## Results

**Figure 2.**
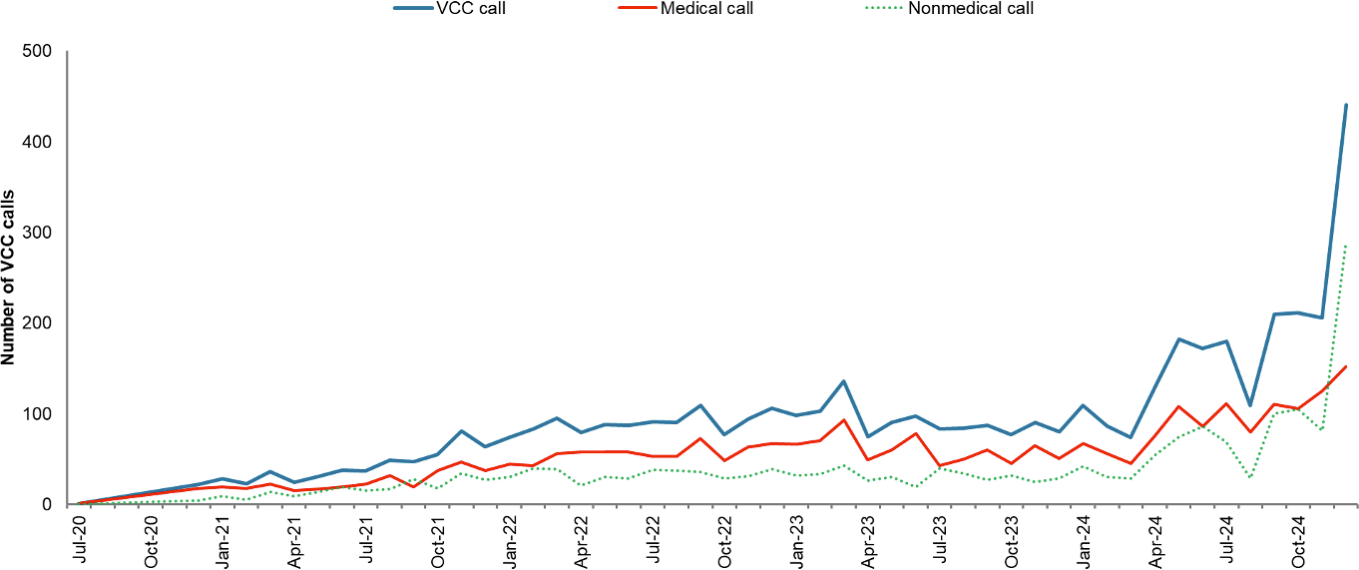
Monthly Virtual Care Centre (VCC) call volumes from July 2020 to December 2024. Call volumes increased steadily, corresponding with the progressive scaling of services across the National University Health System (NUHS) cluster and care settings. The surge in December 2024 reflected the integration of the pre-existing Regional Health System (RHS) Community Care Team (CCT) helpline into the VCC service. Calls to the RHS CCT were directed by VCC care managers (CMs) to the appropriate team. These were classified under VCC for operational reasons, as VCC CMs were the first point-of-contact.

Between July 2020 and December 2024, the VCC received a total of 4857 calls (Figure 2), with the average monthly call volume increasing from about 30 calls per month in late 2020 to over 200 per month by late 2024. This trend corresponds with the progressive scaling of VCC services. A marked increase in all calls in December 2024 coincided with the merger of the VCC helpline and a pre-existing line previously operated by the Regional Health System Community Care Team (CCT).

Of the total, 2879 (59.3%) calls were medical in nature, indicating a need for direct intervention or consultation by VCC clinicians. Most (n=2200, 76.4%) of these calls pertained to medical symptoms, including issues with medical devices such as nasogastric tubes and indwelling catheters (Table 2). This suggests a role for the VCC in guiding caregivers on troubleshooting these devices and, where necessary, coordinating nurse dispatch for further adjustments in the patient’s home.

**Table 2.** Demographic characteristics and call outcomes of medical calls, including caller demographics, call type, day or time patterns, call disposition, emergency department (ED) avoidance rates, triage accuracy, and adverse outcomes.

Variable	Value
Medical calls, n	2879
Call type, n (%)	
Medical symptoms, including NGT^a^ and IDC^b^ issues	2200 (76.4)
Medication-related questions (dosage, timing, effects)	584 (20.3)
Medication side effects	95 (3.3)
Demographics^c^	
Age (y), mean (SD)	70.51 (18.8)
Male, n (%)	1284 (44.6)
Female, n (%)	1595 (55.4)
Call received day of week, n (%)	
Monday AM	423 (14.7)
Monday PM	332 (11.5)
Tuesday AM	296 (10.3)
Tuesday PM	283 (9.8)
Wednesday AM	235 (8.2)
Wednesday PM	282 (9.8)
Thursday AM	238 (8.3)
Thursday PM	294 (10.2)
Friday AM	217 (7.5)
Friday PM	279 (9.7)
Call disposition, n (%)	
Against medical advice	17 (0.6)
Refer to community care team	21 (0.7)
Direct admission (NUHS@Home^d^ or Institution)	94 (3.2)
Direct to 995 or ED^e^	397 (13.8)
Activation of first responder	32 (1.1)
Intervention and co-manage with other specialist	1834 (63.7)
New or earlier outpatient appointment	139 (4.8)
Refer to primary care (GP^f^ or NUP^g^)	179 (6.2)
Transfer to pharmacy	157 (5.5)
Refer to temporary short-term care facility	9 (0.3)
ED avoidance, n (%)	
ED avoidance within 72 hour of call resolution	1990 (69.1)
Triage accuracy, n/N (%)	
Matched triage (VCC^h^ directed caller to ED; caller went to ED within 72 hours of call resolution, and was subsequently admitted to hospital)	148/397 (37.3)
Overtriage (VCC directed caller to ED; caller went to ED within 72 hours of call resolution but was subsequently discharged from ED)	157/397 (39.6)
Undertriage (VCC did not direct caller to ED, recommend inpatient admission, or transferred caller to pharmacy, caller went to ED within 72 hours of call resolution, and was subsequently admitted to hospital)	110/2232 (4.9)
Adverse events, n (%)	
Number of ICU^i^ or HD^j^ admissions within 72 hours of call resolution	0 (0)
Number of mortality cases within 14 days	29 (1)
Number of mortality cases within 30 days	66 (2.3)

a NGT: nasogastric tube.

b IDC: indwelling catheter.

c These are the demographics of callers at all call events. Some callers might call multiple times, and their age at all call events is included in the calculation of the average. The same applies to gender.

d NUHS: National University Health System.

e ED: emergency department.

f GP: general practitioner.

g NUP: National University Polyclinic.

h VCC: Virtual Care Centre.

i ICU: intensive care unit.

j HD: high dependency.

The patient population was predominantly elderlyolder adults, with a mean age of 70 years (SD 18.8) and slightly more female (n=1595, 55.4%), aligning with national data that older adults are disproportionately frequent users of acute services in Singapore [7,9]. Calls were distributed evenly across weekdays, with a slight peak on Mondays, which may reflect symptom build-up or delayed accessing routine care over the weekend.

Nearly two-thirds (n=1834, 63.7%) of the medical calls were managed virtually, either directly by VCC physicians or in comanagement with other specialists (Figure S2 in Multimedia Appendix 3; Table 2). This suggests that most calls were resolved without the need for in-person care. The VCC’s virtual resolution rate is comparable to other telehealth urgent care services, which report a resolution rate of 60% to 70% [20,24,33]. Other calls were appropriately directed to primary care providers, such as GPs or NUHS Polyclinics (n=179, 6.2%), pharmacy services (n=157, 5.5%), or outpatient appointments (n=139, 4.8%). The overall escalation rate to EDs (n=397, 13.8%) was lower than that reported by Australia’s Metro North Virtual ED (29.6%) [20] but comparable to the 6.3% off-site escalation rate reported by Spain’s Quirónsalud program [23]. Some (n=17, 0.6%) patients declined escalation to ED, NUHS@Home, or direct institutional admission, despite clinical recommendations. These decisions might be influenced by individual preferences, fear of long ED waits, cost considerations, or confidence in managing symptoms at home. Future evaluation of these cases will be essential for safety monitoring.

**Figure 3.**
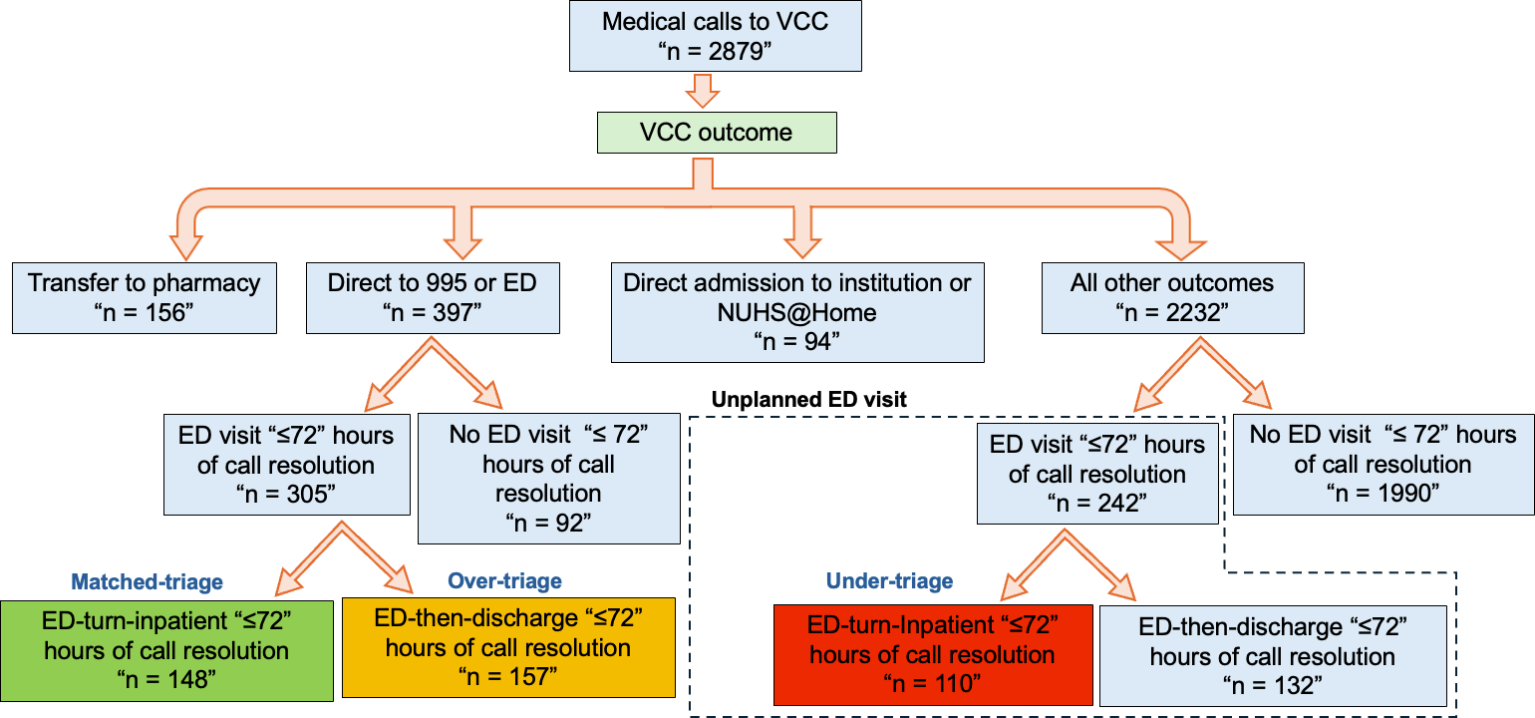
Triage accuracy and emergency department (ED) avoidance. Proportion of calls managed virtually, ED referral rates, and triage accuracy including matched-triage, overtriage, and undertriage outcomes. NUHS: National University Health System; VCC: Virtual Care Centre.

A total of 69.1% (n=1990) of the calls were not followed by an ED visit within 72 hours of call resolution (ED avoidance; Figure 3, Table 2). Among those referred to the ED, 37.3% (148/397) visited the ED within 72 hours of call resolution and were subsequently admitted to the hospital, demonstrating the appropriate identification of higher-acuity cases (matched triage). Overtriage, where callers were directed to the ED and presented at the ED within 72 hours of call resolution but were subsequently discharged from the ED, was moderate at 39.6% (157/397), while undertriage was low, at 4.9% (110/2232).

Outcome metrics indicate that the VCC’s tele-triage model achieved positive safety outcomes. Importantly, adverse outcomes were infrequent. No high dependency (HD) or ICU admissions occurred within 72 hours of call resolution. Mortality rates were 1.0% and 2.3% within 14 and 30 days, respectively (Table 2). Similar results have been reported across tele-triage and virtual urgent care evaluations, demonstrating reduced ED use without increased risks of admission, ICU use, or mortality. In the US Veterans Affairs tele-EC program [34], ED visits declined with no increase in admissions or 30-day mortality; Ontario’s VUC [35] showed hospitalization and death rates comparable to in-person ED care; and Australia’s Virtual ED [20] reported only 1.2% unexpected admissions within 48 hours, with no serious adverse outcomes.

Post-call surveys were conducted in February 2023 and October to November 2024, with 91% (79/87) of respondents rating the VCC service 8 or higher out of 10 (Figure S3 in Multimedia Appendix 4), indicating high levels of user satisfaction.

## Discussion

This study describes the implementation and early outcomes of the NUHS VCC, a medical helpline designed to reduce unnecessary ED visits and support community-based acute care for residents living in the western region of Singapore. The data from July 2020 to December 2024 represent the initial implementation and expansion of the service across the NUHS cluster.

### Summary of the Main Findings

More than 76% (2200/2879) of medical inquiries were regarding medical symptoms, with the rest being medication-related inquiries. Callers were predominantly elderlyolder adults, with a mean age of 70.51 (SD 18.8) years. ED avoidance and accuracy of triage were assessed by comparing VCC call disposition with subsequent ED utilization within 72 hours of call resolution. These metrics were formulated with reference to prior virtual emergency care evaluations [33]. From service inception through December 2024, the 72-hour ED avoidance rate was 69.1%. Triage performance demonstrated matched-triage, overtriage, and undertriage rates of 37.3%, 39.6%, and 4.9%, respectively. Safety metrics were reassuring, with no ICU or HD admissions within 72 hours of call resolution and low 14-day and 30-day mortality rates.

### Detailed Discussion of the Findings, Including Interpretations, Implications, and Comparisons to Existing Literature

The data demonstrate VCC’s contribution to reducing unnecessary ED visits, with most cases managed without in-person ED attendance. Nearly two-thirds of medical calls were resolved virtually, and only <15% were directed to the ED. The combination of a 69.1% ED avoidance rate, low undertriage, zero ICU/HD admissions within 72 hours, and low 14-day and 30-day mortality rates supports the clinical safety of this tele-triage model during the study period. It is important to recognize that ED avoidance and triage accuracy were defined using operational criteria based on counts of events within a fixed 72-hour window. These measures do not account for the clinical relationship between the original call and subsequent ED visits or hospital admissions. For example, a caller might have called for a mild complaint, been advised to monitor symptoms at home, and might later develop an unrelated acute condition requiring admission within 72 hours. Under the current framework, this case would have been classified as undertriage, even though the later presentation might not have been causally related to the initial call. Examining subsequent ED diagnoses and causes of mortality, where relevant, would allow a more precise assessment of triage performance.

As a medical helpline, the VCC primarily functions as an advisory service. Patients retain discretion as to whether to follow recommendations to attend the ED. The ED avoidance observed in our cohort is broadly comparable to diversion rates reported in other virtual ED models. Similar proportions of patients managed virtually with low rates of unexpected admission have been described in the Australian virtual ED model [20], and comparable diversion rates were reported by Sri-Ganeshan et al [33]. In contrast, earlier evaluations of large-scale telephone triage systems, such as NHS 111, did not demonstrate reductions in ED attendance and were associated with a small increase in ambulance dispatches [24]. These differences may reflect variations in service design, particularly the availability of direct clinical assessment and integration with hospital systems.

Beyond diverting cases from the ED, the VCC facilitates referrals to services such as NUHS@Home, direct hospital admission, specialist clinics, and community teams. In the context of Singapore’s aging population and rising chronic disease burden [7-10], such services may help in the appropriate use of hospital resources while maintaining access to timely medical advice. Although around one -fifth of calls were medication related, only 5.5% were transferred to the pharmacy. This demonstrates that clinical assessment by physician or nurses is required for most of these cases rather than dispensing advice alone.

The VCC service was predominantly used by elderlyolder patients, most of whom were female. This pattern is consistent with broader health care utilization trends among older adults in Singapore. Similar trends have also been reported in telephone medical helplines internationally, where elderlyolder women were frequent users [36]. While our data do not allow for conclusions regarding the underlying reasons for these calls, working more closely with community or social support services may further strengthen support for vulnerable older populations. A slight increase in call volume was observed at the start of the week, which is typical of ED visits and described as the “fresh start effect” [37,38]. This has operational implications, as staffing levels could be calibrated to accommodate predictable fluctuations in demand early in the week.

### Limitations and Implementation Challenges

Pilot outcomes suggest that the NUHS VCC model is operationally feasible and clinically practical, with many urgent cases managed virtually and the potential to reduce unnecessary ED visits. It also addresses a gap in post-discharge and community-based transitional care by offering timely advice and escalation pathways. Nevertheless, several limitations and implementation challenges must be acknowledged.

First, VCC is not yet fully integrated across all levels of care, despite being situated within the NUHS cluster. Coordination with primary care, community services, and other external providers remains manual and dependent on individual clinicians. This constrains the model’s ability to support seamless continuity beyond the initial triage episode. Moreover, VCC primarily addresses acute episodic needs and does not encompass chronic disease management or long-term follow-up. Its reliance on telephone (audio-only) consultations limits detailed visual assessments of patients.

Second, the ED avoidance rate reported here was based on whether callers who were directed to an ED presented within 72 hours of call resolution. Callers retained discretion over whether to follow the VCC recommendation. Not all calls were validated by a physician, as CMs and nurses were empowered to direct callers to an ED depending on the severity of their reported symptoms. Therefore, the ED avoidance and triage accuracy rates in this study might not be directly comparable to those of other virtual EDs with different care models (eg, Australia’s Queensland’s Metro North Virtual ED program) [20], where patients had to call the hotline before presenting at an ED. Since December 2024, as part of the post-call survey, patients were asked whether they would have visited an ED if they had not called VCC. An ED avoidance rate based on that survey question would differ from the one reported here. Furthermore, since the data were extracted from the NUHS EMR system, callers who visited EDs at hospitals in other clusters would not be captured in the database.

Third, NUHS VCC is still in its early evaluation phase, with limited evidence on long-term outcomes, patient experience, cost-effectiveness, and system-wide impact. As a retrospective study, the findings presented here are limited by residual confounding and incomplete patient-level data. Prospective studies are required to validate its effectiveness and inform scalability, which will also depend on workforce capacity, sustainable funding, and integration with the national health IT infrastructure.

The implementation of the VCC also presented a few challenges. Defining the appropriate clinical protocols for prehospital telephone triage required the adaptation of in-person triage frameworks to a telephonic context. A tiered escalation protocol tailored to the VCC was developed to enable CMs to identify emergencies through structured symptom checklists and vital sign thresholds. Physicians reviewed triage assignments and provided feedback during the pilot phase, allowing iterative refinements to improve safety and accuracy.

Encouraging adoption among patients and community providers was another challenge. Targeted outreach strategies were implemented to drive engagement and visibility. Patients received brochures and reminders via verbal communication and SMS at various discharge points, encouraging the use of the helpline instead of visiting the ED. Engagement sessions with NUHS Primary Care Network GPs and nursing home partners promoted tele-collaboration and clarified referral workflows. Public awareness was further strengthened through national media coverage featuring the VCC’s benefits and patient testimonials. Continuous reinforcement remains essential to firmly establish the VCC as a first-line option for noncritical acute care.

Sustaining a virtual care service at scale presents considerable workforce challenges. Recruiting and retaining qualified personnel willing to work shifts, including nights and weekends, has been difficult. The evolving digital landscape also requires staff who are skilled in remote workflows and virtual care technologies. Although telephony call management platforms have been introduced to support off-site flexibility, maintaining adequate staffing levels remains a key risk to operational viability. Securing sustainable funding for the program is another long-term challenge.

Engaging diverse stakeholders, including hospital specialists and administrators, as well as community-based general practitioners and nursing home staff, required the alignment of governance frameworks and shared goals. Stakeholders varied in their familiarity with virtual care models, and institutional priorities often delayed adoption. This was particularly evident when onboarding new clinical departments and expanding services beyond AH.

In addition to operational challenges, ethical, equity, and safety considerations were integrated into the VCC’s implementation and evaluation. The service uses operational data under institutional governance oversight. Structured triage protocols and escalation pathways were developed to minimize undertriage risk, with ongoing monitoring of ICU admissions and short-term mortality as safety indicators. Although the telephone-based model may reduce digital literacy barriers compared with app-based platforms, individuals without awareness of the service or access to telecommunication resources may remain underrepresented. Continued monitoring is therefore necessary to mitigate potential bias and support equitable access.

Together, these challenges highlight the operational complexity of implementing a virtual acute care model at scale. Overcoming them required not only innovation and agility but also sustained inter-organizational collaboration, investment in workforce and digital infrastructure, and deliberate efforts to reshape care-seeking behaviors among providers and the public.

### Conclusions and Future Directions

The NUHS VCC represents an early step toward integrating virtual triage within a regional acute care framework. This pilot evaluation suggests that a clinician-led medical helpline can manage a substantial proportion of noncritical acute medical inquiries virtually, with low adverse outcomes and the potential to reduce unnecessary ED attendance. Within a hospital cluster, the model has demonstrated operational feasibility and highlights the role of centralized tele-triage in improving access to timely medical advice. As demand for emergency and urgent care continues to rise, particularly in aging populations, services such as the VCC may help guide lower-acuity cases to appropriate care settings while preserving ED capacity for more serious conditions. In this context, structured tele-triage can function as an additional layer of access before hospital presentation.

As the VCC expands, ongoing evaluation will be important to assess longer-term clinical outcomes, patient experience, cost-effectiveness (eg, cost per ED visit avoided), and overall system impact. Prospective studies incorporating diagnostic-level validation of triage accuracy and broader data linkage across health care clusters would strengthen the assessment of safety and effectiveness. Additional work is also needed to evaluate whether virtual triage influences downstream healthcare utilization patterns beyond the 72-hour window examined in this study.

Continued development should focus on deeper integration with primary and community care, improving digital interoperability, and ensuring sustainable workforce models. With careful refinement and evaluation, VCC may contribute meaningfully to how urgent care is accessed and delivered within Singapore’s public health system.

## Supplementary material

10.2196/86556 Multimedia Appendix 1 Timeline of National University Health System (NUHS) Virtual Care Centre (VCC) implementation and service expansion. The figure outlines the phased rollout of the VCC from its initial pilot at Alexandra Hospital in 2020 to wider expansion across NUHS cluster institutions, including the Primary Care Network, National University Polyclinics, NUHS@Home, Advanced Internal Medicine (AIM), Ng Teng Fong General Hospital (NTFGH), National University Hospital (NUH), and NUHS. Key milestones include general practitioner access (2023) and inclusion of nursing teams (2024).

10.2196/86556 Multimedia Appendix 2 Disposition of medical calls managed by Virtual Care Centre (VCC) doctors from July 2020 to December 2024. Nearly two-thirds of calls were resolved virtually through intervention or comanagement with other specialists. Other cases were redirected to primary care, escalated to the emergency department (ED), or admitted as inpatients. Singapore Civil Defence Force (SCDF/995);. GP: general practitioner; NUP:, National University Polyclinic;. GP, general practitioner.†Indicates cases where the VCC doctor directed a patient to the ED or for direct admission to NUHS@Home (hospital-at-home) or institution, but the patient refused.

10.2196/86556 Multimedia Appendix 3 Patient satisfaction with Virtual Care Centre (VCC) services based on postcall surveys conducted in February 2023 and October to November 2024. Respondents (n=87) were asked to rate their overall satisfaction with the VCC on a scale from 1 (very dissatisfied) to 10 (very satisfied). A total of 91% rated the service 8 or higher. These surveys were conducted by VCC staff via telephone during routine follow-up calls.

10.2196/86556 Multimedia Appendix 4 A 4-tier Virtual Care Centre (VCC) telephone triage protocol is used to prioritize medical call responses. The protocol was adapted from established emergency department (ED) triage frameworks to guide care managers (CMs) in stratifying call urgency. Each tier defines symptom categories, red flags, and corresponding response times. CMs initially performed triage during the pilot phase; acute care-trained nurses were formally integrated into frontline triage from 2024.

## Abbreviations

AH: Alexandra Hospital
CCT: Community Care Team
CM: case manager
ED: emergency department
EMR: electronic medical record
GP: general practitioner
HD: high dependency
ICU: intensive care unit
NUHS: National University Health System
STROBE: Strengthening the Reporting of Observational Studies in Epidemiology
VCC: Virtual Care Centre
VUC: Virtual Urgent Care
