# Supplementary figures and images for: Regional Virtual Acute Care Helpline in Singapore at a National University Health System Virtual Care Centre: Retrospective Study
Source: JMIR Form Res. 2026 May 13;10:e86556. doi: 10.2196/86556 (PMC13170088; doi:10.2196/86556)

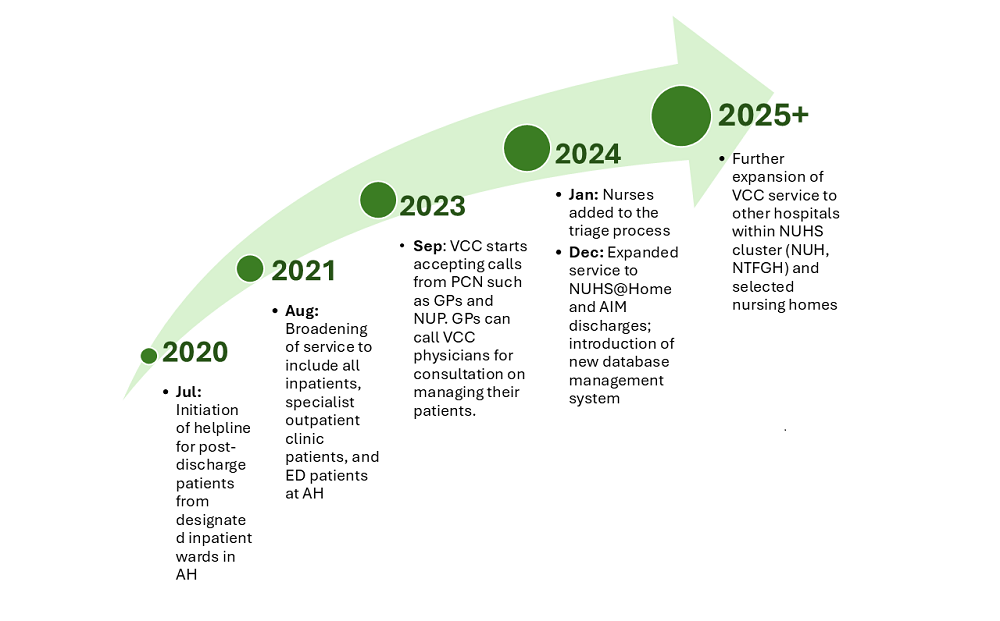

Supplement: Multimedia Appendix 1 [file formative-v10-e86556-s001.png]

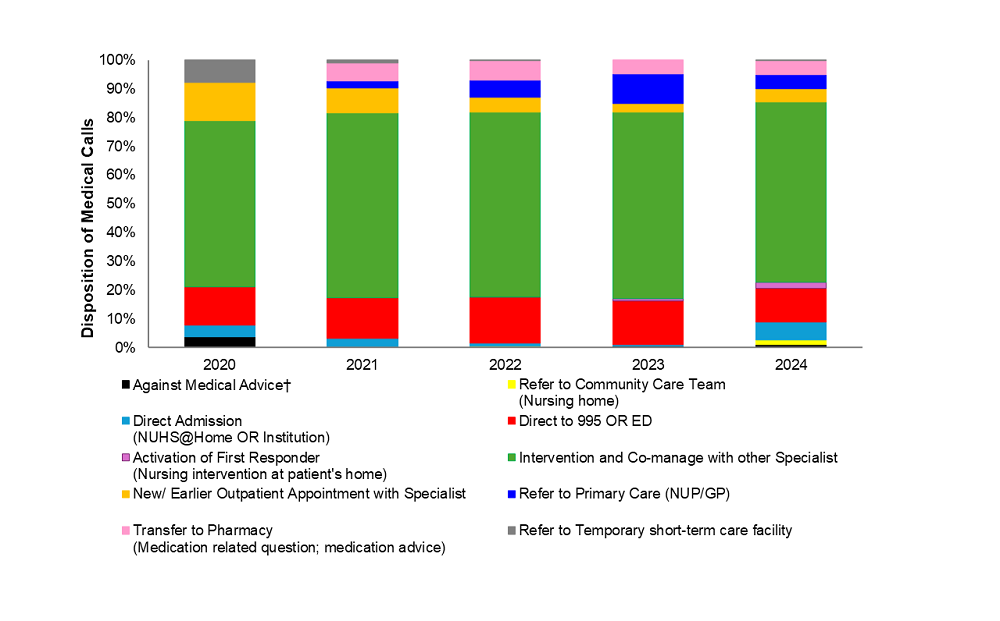

Supplement: Multimedia Appendix 2 [file formative-v10-e86556-s002.png]

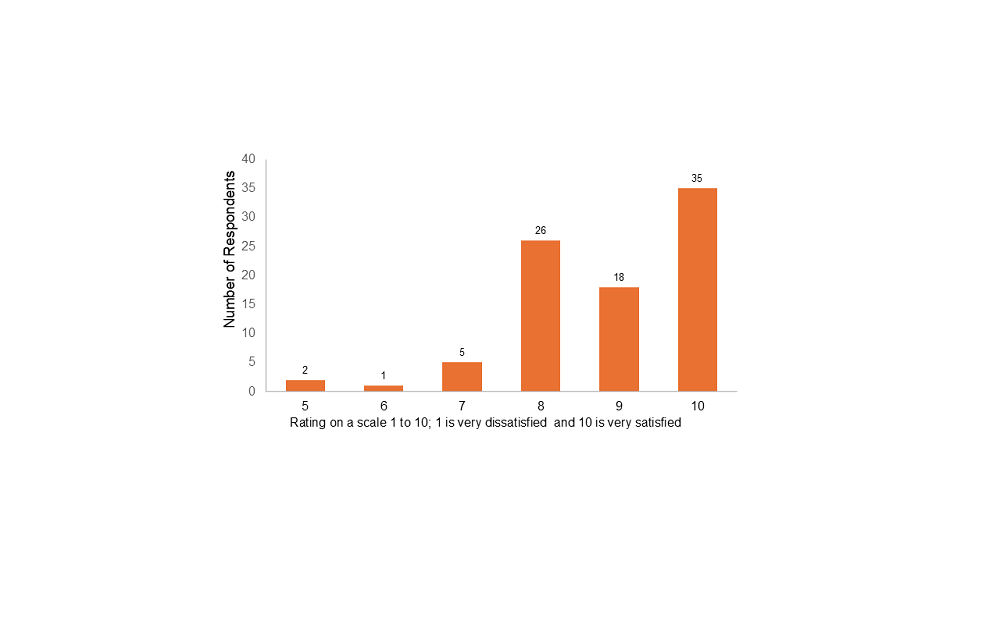

Supplement: Multimedia Appendix 3 [file formative-v10-e86556-s003.png]

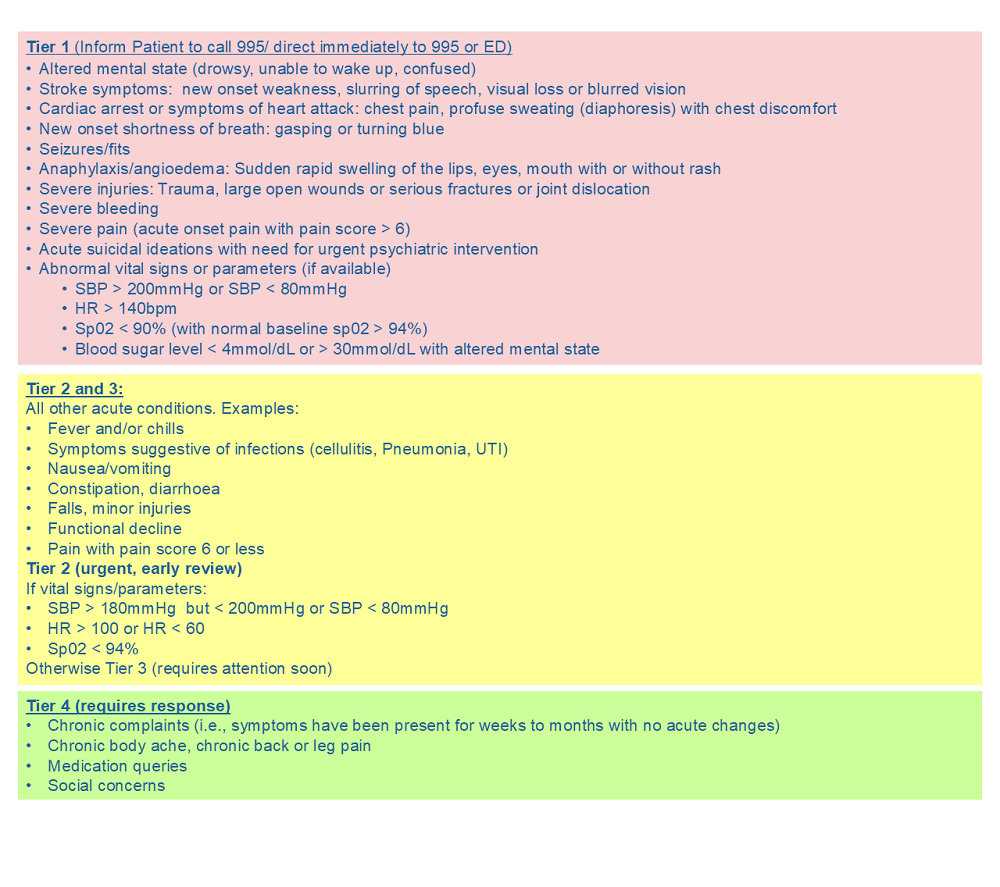

Supplement: Multimedia Appendix 4 [file formative-v10-e86556-s004.png]
